# Activating clinical trials: a process improvement approach

**DOI:** 10.1186/s13063-016-1227-2

**Published:** 2016-02-24

**Authors:** Diego A. Martinez, Athanasios Tsalatsanis, Ali Yalcin, José L. Zayas-Castro, Benjamin Djulbegovic

**Affiliations:** Department of Emergency Medicine, Johns Hopkins University, 5801 Smith Avenue Baltimore, Baltimore, 21209 MD USA; USF Health Program for Comparative Effectiveness Research, Department of Internal Medicine, University of South Florida, 12901 Bruce B. Downs Blvd., MDC 27, Tampa, FL 33612 USA; Department of Industrial and Management Systems Engineering, University of South Florida, 4202 East Fowler Avenue, Tampa, 3360 FL USA; H. Lee Moffitt Cancer Center and Research Institute, 12902 USF Magnolia Drive, Tampa, 33612 FL USA

**Keywords:** Clinical trials, Time factors, Quality improvement

## Abstract

**Background:**

The administrative process associated with clinical trial activation has been criticized as costly, complex, and time-consuming. Prior research has concentrated on identifying administrative barriers and proposing various solutions to reduce activation time, and consequently associated costs. Here, we expand on previous research by incorporating social network analysis and discrete-event simulation to support process improvement decision-making.

**Methods:**

We searched for all operational data associated with the administrative process of activating industry-sponsored clinical trials at the Office of Clinical Research of the University of South Florida in Tampa, Florida. We limited the search to those trials initiated and activated between July 2011 and June 2012. We described the process using value stream mapping, studied the interactions of the various process participants using social network analysis, and modeled potential process modifications using discrete-event simulation.

**Results:**

The administrative process comprised 5 sub-processes, 30 activities, 11 decision points, 5 loops, and 8 participants. The mean activation time was 76.6 days. Rate-limiting sub-processes were those of contract and budget development. Key participants during contract and budget development were the Office of Clinical Research, sponsors, and the principal investigator. Simulation results indicate that slight increments on the number of trials, arriving to the Office of Clinical Research, would increase activation time by 11 %. Also, incrementing the efficiency of contract and budget development would reduce the activation time by 28 %. Finally, better synchronization between contract and budget development would reduce time spent on batching documentation; however, no improvements would be attained in total activation time.

**Conclusion:**

The presented process improvement analytic framework not only identifies administrative barriers, but also helps to devise and evaluate potential improvement scenarios. The strength of our framework lies in its system analysis approach that recognizes the stochastic duration of the activation process and the interdependence between process activities and entities.

**Electronic supplementary material:**

The online version of this article (doi:10.1186/s13063-016-1227-2) contains supplementary material, which is available to authorized users.

## Background

Clinical trials are the means to transform scientific discovery into medical utility, and they are designed to attain evidence on safety, efficiency, and effectiveness of investigated interventions. Clinical trials have been credited with major therapeutic and diagnostic discoveries and improvement in health outcomes including life expectancy [1, 2]. However, despite these well-documented medical advances, clinical trials have been challenged for exhibiting less than efficient administrative procedures preceding trial recruitment that are costly [3–7], complex [8–11], and time-consuming [5, 7, 12–14].

Evidence to these claims include published work such as a 2005 review demonstrating that activating a trial (i.e., allow for patient accrual) requires on average 32 person hours per patient accrued, which in 2005 was roughly translated into a cost of US$1,500 per patient enrolled [15]. Since 2005 costs associated with trial activation have risen considerably, reaching approximately US$50,000 per trial, regardless of the number of patients accrued [7]. As a result, the costs of clinical research have increased between 31 and 88 % from 2008 to 2013, depending on trial phase [16]. This sizable amount is exonerated if we consider that the administrative process associated with trial activation in academic medical settings includes up to 30 different activities, involves up to 11 participants, and lasts on average, from 44 to 172 days [6, 8, 17, 18]. Such complexity translates into delayed trial activation, which affects patient enrollment goals [19, 20] and diminishes the usefulness of trials by failing to attain evidence of investigated interventions on time [21].

Several authors have scrutinized the efficiency of trial activation. Although early studies concentrated on identifying administrative barriers and provided little to no recommendations for improvement [6, 8–10], more recent studies have shed light on strategies to improve efficiency by using a combination of six sigma and lean thinking methods that remove non-value-added activities in the administrative process [14, 17, 18, 22]. These studies are of importance for effective trial management and resource utilization; however, their results might not be applicable across all clinical research settings and thus, triggering the need for models that support process improvement decision-making. Modeling frameworks providing insights as to where efficiency improvements can be attained before implementing devised improvement scenarios can help to strategize resources and design a better trial activation process. To minimize the time and resources needed to move trials from concept development to patient enrollment, it is necessary to have a profound understanding of the activation process and of the potential impact of devised improvement scenarios. Integrating concepts of business process modeling in the analysis of operational efficiency of trial activation, as others have successfully accomplished (e.g., [23, 24]), is expected to provide useful insights to streamline the administrative process.

This paper presents a process improvement framework that relies on formal techniques such as process mapping, social network analysis (SNA), and discrete-event simulation (DES). Our work expands on previous research by incorporating the use of SNA and DES to create an analytic framework to support process improvement decision-making. The combination of these techniques allows for an extensive understanding of the potential impact of devised improvement scenarios. The presented framework is applied to an illustrative case, the Office of Clinical Research (OCR) at the University of South Florida (USF) in Tampa, Florida, and a number of interesting results and managerial insights are discussed for clinical research administrators. The combination of these techniques formed a novel approach that can be used to understand drivers of process performance, and to ultimately improve overall efficiency of the opening of clinical trials.

## Methods

To assist the reader, we define here the terminology used throughout this paper. We use the terms administrative process, or process, to denote the overall series of administrative activities required to move a trial from concept development to trial activation. Trial activation, or activation, is achieved when the trial is allowed to enroll patients. The term “sub-process” refers to a distinct section of the administrative process. As explained in the results section, we have identified five sub-processes: Initial Preparation; Contract Negotiation; Budget Negotiation; Preparation for Western Institutional Review Board submission; and Final Preparation, which includes the final approvals by the principal investigator (PI), the USF’s Division of Sponsored Research and the trial sponsor. Activity denotes the smallest amount of work within a sub-process. Each sub-process is comprised of a finite set of activities. A participant is any person or entity with a role in an activity. Activation time is time duration from the OCR’s study protocol receipt to trial activation. Idle time is the time spent by each trial from the end of a sub-process until the start of the next one (e.g., waiting for approval signatures or paperwork delays). Finally, queue length denotes the average number of trials waiting to be processed at each sub-process.

### Study setting and study team

This study was conducted at the OCR of the University of South Florida in Tampa, Florida [25]. The study team was comprised of four process improvement specialists from the USF’s College of Engineering, a clinical research administrator from the OCR, and a physician from the USF’s College of Medicine.

### Dataset

We searched the OCR database for all industry-sponsored trials initiated and activated within the period of July 2011 and June 2012 (see Additional file 1). These trials included extensive information on duration of each activity and on interactions between participants. For each trial, we extracted names and time stamps of all activities pertinent to trial activation such as receipts, submissions, and approvals of regulatory documentation. We also extracted time stamps and volume of interactions between participants from communication logs, written documentation, and email communication.

### Process mapping and timing analysis

We used value stream mapping (VSM) to create an overview of the administrative process associated with trial activation, starting from concept development and ending with trial activation. By using VSM we can identify, and potentially eliminate or modify, non-value-added activities in the administrative process [5–10]. These activities may represent waste of resources such as time, money, and effort. According to Dilts ([8], p. 4549), a non-value-added activity is an activity that does not contribute to the research integrity, patient safety, or usefulness of the clinical trial such as paperwork delays, batching of documentation, or paper movements. We validated the integrity and accuracy of the process maps with senior OCR personnel. Additionally, activity time stamps were used to compute the duration of each sub-process and of the overall process. We calculated descriptive statistics to measure central tendency (i.e., average and median) and dispersion (i.e., standard deviation and interquartile range).

### Social network analysis

Descriptive models relying on process mapping and timing analysis have been used by previous authors to study the administrative process [6, 8–10]. Despite their usefulness, such models are not primarily designed to capture any information regarding participant interactions. Therefore, potentially significant information regarding the social environment at which the administrative process is performed is overlooked. As a result, strategies aiming at improving process efficiency may be misdirected. The question we aim to answer is: Who needs to be involved in the improvement process? By using SNA, we can analyze the interactions between OCR and other participants and identify those who must be involved in the improvement process.

SNA is developed to understand social networks and reveal information regarding participants and their interactions. It has been extensively used to explain phenomena such as scientific interaction [26] and information exchange [27]. A social network consists of a set of nodes and ties. In this study, a node represents a participant in the administrative process and a tie represents an interaction between participants. The direction of a tie denotes the direction of an interaction. For instance, a PI emailing a research protocol to the OCR for revision would be classified as an interaction from PI to OCR.

We focused the analysis on centrality measures, which show the importance and influence of a participant in the process. We hypothesize that a participant’s centrality is highly correlated to the participant’s workload and, therefore, improvement strategies should target the most central participants. The centrality measures we used are those of degree, closeness, betweenness, and Bonacich’s Power Index [28]. *Degree* is a measure of volume of interactions that a participant has with others. High out-degree indicates that a participant initiates many interactions (i.e., sends documents to many other participants) and a high in-degree indicates that a participant receives many interactions (i.e., receives documents from many other participants). *Closeness* is a measure of distance between participants. High out-closeness implies that a participant can reach others in few steps. High in-closeness implies that others can reach a participant in few steps. We interpret closeness as the distance that a document has to travel before it reaches the intended receiver. Presumably, small closeness denotes faster flow of information. *Betweenness* is a measure of how important a participant is in connecting other participants. Participants with high betweenness are very important to the network to ensure connectivity; without it, some interactions may never occur. Finally, Bonacich’s Power Index measures the power of each participant in the network based on its capacity to connect with others. High Bonacich’s Power Index indicates that the neighborhood of a participant is not well-connected. The rationale behind this index is that a participant is more powerful if it is connecting other participants that are not connected among them. We used NetDraw [29] to compute centrality measures and to draw social network diagrams.

### Simulation model

The outputs of process mapping, timing analysis, and SNA can offer descriptive information of the clinical trial activation process. The process map described the entities, events, and resources needed to complete each activity, and the timing analysis provided details on the time between arrival of trials to the OCR as well as the processing times in each sub-process. This information is practically an observation of the current status of the system and ultimately can be used to assist in devising scenarios for process improvement. These scenarios can be implemented in a trial-and-error manner (i.e., implementing without prior evidence of improvement), or further studied using computer simulations. DES is used to simulate and compare the validity, efficiency, and effectiveness of various scenarios. Compared to other modeling techniques such as queuing theory, DES models are more flexible, provide more information on results, and are easier to build [30].

The administrative process to activate a clinical trial can be seen as a sequence of discrete events with stochastic duration in which regulatory documentation (i.e., entities) travels through several activities, decisions, and loops until all regulatory committees, sponsors, and PIs declare the clinical trial ready for patient enrollment. There are uncertainties of process performance and system workload, as well as dependence between process activities and regulatory documentation. Such dependency is seen, for example, when the trial activation can only continue if both the non-disclosure agreements and the trial budget are agreed upon. In the presence of operational data, DES represents a suitable and more flexible modeling approach than others [31]. Although decision-makers might be skeptical about the validity of a DES model, there is no other way to provide a realistic estimate of the impact of improvement scenarios before actual implementation. Other modeling techniques, such as decision trees, impose rigid structures based on mutually exclusive events (i.e., process activities) and they do not explicitly consider time. An alternative approach that considers time is Markov chains; however, this retains the structural rigidity making it difficult to represent the administrative process. DES is preferable over decision trees and Markov chains because there is no assumption of independence between entities [32]. In the administrative process associated with trial activation, the entities are regulatory documentation seeking for approval; therefore, incorporating interactions in the modeling approach becomes a necessity. In addition, in DES models full probabilistic analysis take place naturally. All inputs can be defined using any probability density function allowing the incorporation of both real variation (e.g., in the processing time of a trial budget) and uncertainty (e.g., in the effect of each improvement scenario). Hence the use of DES is preferable over other modeling techniques to aid the process improvement decision-making.

DES requires definition of entities, events, and resources. In our simulation model, which is described in detail in the Additional file 2, entities represent regulatory documentation required for trial activation (e.g., non-disclosure agreements and clinical trial budget); events represent the preparation, revision, and approval of such documentation; and resources represent the personnel working on such events. The performance measures utilized to assess process efficiency are activation time, idle time, and queue lengths.

Different scenarios with the potential of affecting system performance were devised. These scenarios were built using resource capacity and processing time distributions as input variables. All simulation analyses were performed using Arena [33]. Time between arrivals and processing times were modeled with theoretical probability density functions. The choice of appropriate density functions was based on minimizing the sum of square errors as described in Table 1. Power analysis showed that 38 replications ensured less than 5 % error in our performance measures. The simulation length was 1 year (365 calendar days) preceded by a warm-up period of 4 years (1460 calendar days).

**Table 1:** Distribution fitting for processing times at each sub-process and activity in the administrative process associated with industry-sponsored clinical trial activation at the University of South Florida. In the simulation software Arena, the default random number stream 10 was used to generate the duration of each sub-process

Subprocess (*activity*)	Squared error	Parameters	Arena random number stream
Initial Preparation			
* OCR sends documents*	-	Uniform (0.5, 1.0)^a^	10
* P&L reviews*	-	Triangular (1.0, 8.0, 21.0)^a^	10
* PI prepares documents*	-	Triangular (1.0, 2.0, 3.0)	10
* P&L reviews*	-	Uniform (0.5, 1.0)	10
Contract Negotiation	0.01	Exponential (28.5)	10
Budget Negotiation	0.01	Exponential (25.0)	10
PI Approval	0.02	Lognormal (5.0, 8.3)	10
DSR Approval	0.02	Lognormal (2.5, 2.8)	10
Sponsor	0.02	Lognormal (6.5, 15.9)	10

*DSR* USF’s Division of Sponsored Research, *OCR* USF’s Office of Clinical Research, *P&L* USF’s Division of Patents and Licensing, *PI* principal investigatorl

^a^Estimated by OCR senior personnel

## Results

### Dataset

We identified 147 clinical trials in the time period of July 2011 and June 2012. We extracted time stamp data of all trials started and completed during the study period, resulting in a final sample of 78 out of 147 trials. A total of 69 trials were excluded from time stamp extraction due to having missing data (*n* = 16), being still in process (*n* = 52), or because they were terminated for reasons beyond the scope of this study (*n* = 1). Data on the frequency of interactions between participants were extracted out of communication logs of those 78 trials. During post-hoc analyses no significant differences in the durations of Contract Negotiation (54.91 versus 57.95, *P* value = 0.6993) and Budget Negotiation (46.3 versus 60.76, *P* value = 0.0712) after adding duration data of the clinical trials still in process. The independent *t* test for samples with equal variance was used to compare differences between groups (see Additional file 2, p. 13).

### Process mapping and timing analysis

**Fig. 1:**
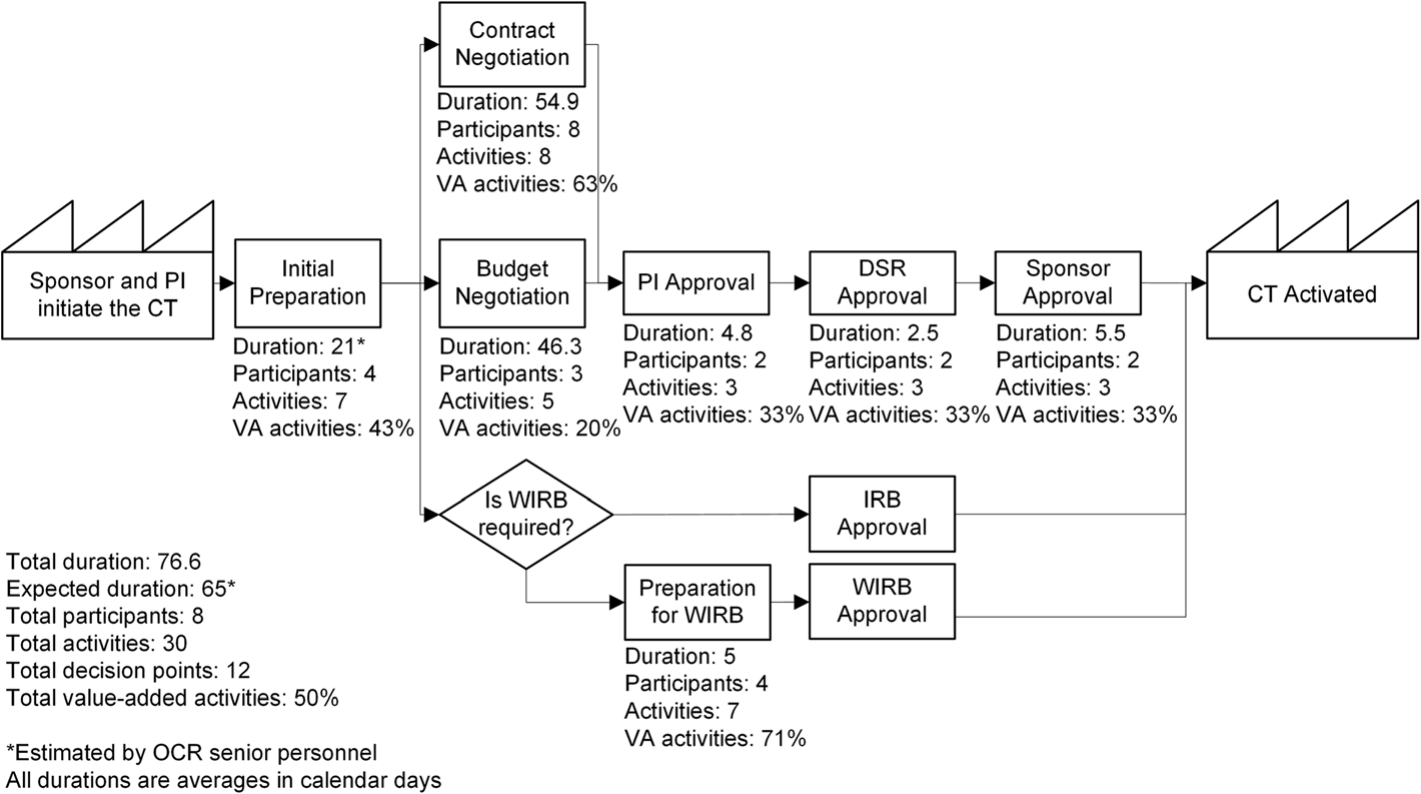
Administrative process associated with industry-sponsored clinical trial activation at the University of South Florida. *CT* clinical trial, *DSR* USF’s Division of Sponsored Research, *IRB* Institutional Review Board, *OCR* USF’s Office of Clinical Research, *PI* principal investigator, *VA* value-added, *WIRB* Western Institutional Review Board. All durations are averages (median) in calendar days. *Estimated by OCR senior personnel

A highly aggregated version of the administrative process associated with trial activation is depicted in Fig. 1 (for a more granular description see Additional file 2: Figure SI2). The administrative process is decomposed into five major sub-processes: Initial Preparation; Contract Negotiation; Budget Negotiation; Preparation for Western Institutional Review Board (WIRB); and PI, USF’s Division of Sponsored Research, and Sponsor Approvals (Final Preparation).

The administrative process lasts on average 76.6 days with a standard deviation of 41.1 days (see Table 2). Contract Negotiation and Budget Negotiation are the most time-consuming sub-processes lasting 54.9 (median = 33, SD = 50.3) and 46.3 (median = 40, SD = 34.6) days on average, respectively. During these two sub-processes and based on conversations with the OCR senior personnel, negotiations between OCR, sponsor, and PI are the most arduous and time-consuming activities. As observed in Fig. 1, Contract Negotiation and Budget Negotiation are parallel sub-processes having different durations. Since both sub-processes must be completed before continuing to the following sub-processes, synchronization issues between these two generate delays in the Entire Process.

**Table 2:** Duration of the administrative process associated with industry-sponsored clinical trial activation at the USF’s Office of Clinical Research (OCR)

Sub-process	Number	Duration in calendar days	
		Average (Median, SD)	IQR (Q1, Q3)
Initial Preparation	-	11^a^	-
Contract Negotiation	69	54.9 (33, 50.3)	58 (19,77)
Budget Negotiation	40	46.3 (40, 34.6)	54 (14, 68)
PI Approval	78	4.8 (4, 4.7)	6 (1, 7)
DSR Approval	78	5.5 (3, 6.2)	9 (0, 9)
Sponsor Approval	78	5.5 (3, 6.2)	9 (0, 9)
Entire Process	78	76.6 (69, 41.1)	58 (46, 101)

*DSR* USF’s Division of Sponsored Research, *IQR* interquartile range, *PI* principal investigator, *SD* standard deviation

^a^Estimated by OCR senior personnel

### Social network analysis for Contract Negotiation and Budget Negotiation

The process mapping and timing analysis showed that Contract Negotiation and Budget Negotiation are the most time- and effort-consuming duties during trial activation. Therefore, we focused the SNA on these two sub-processes and their participants. The question we aim to answer here is: Who needs to be involved in the improvement process?

**Fig. 2:**
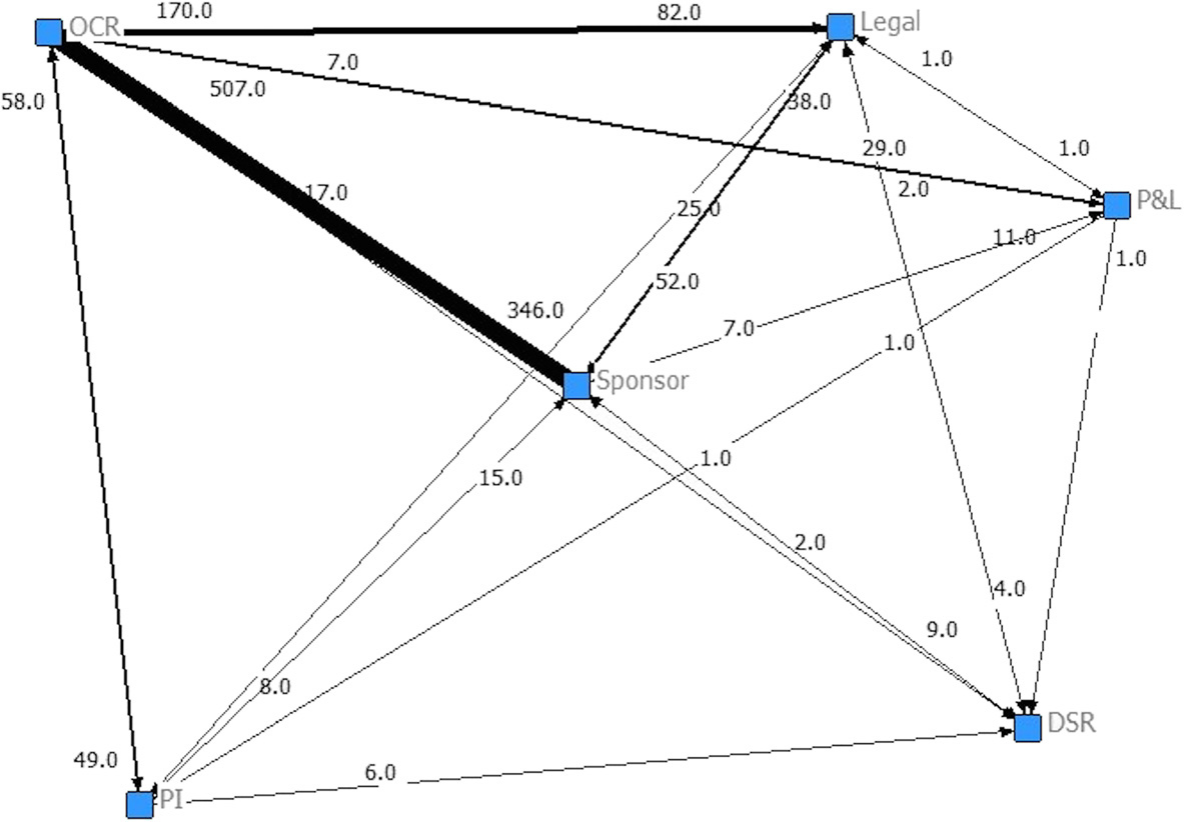
Social network diagram for Contract Negotiation. Each node represents a participant, each tie denotes an interaction between participants, and each tie width denotes the frequency of interactions between two participants. The number closest to the node represents the number of interactions started in the node to others. During Contract Negotiation the majority of interactions occur between OCR, sponsors, and USF Legal. *DSR* USF’s Division of Sponsored Research, *OCR* USF’s Office of Clinical Research; *P&L* USF’s Division of Patents and Licensing, *PI* principal investigator

**Fig. 3:**
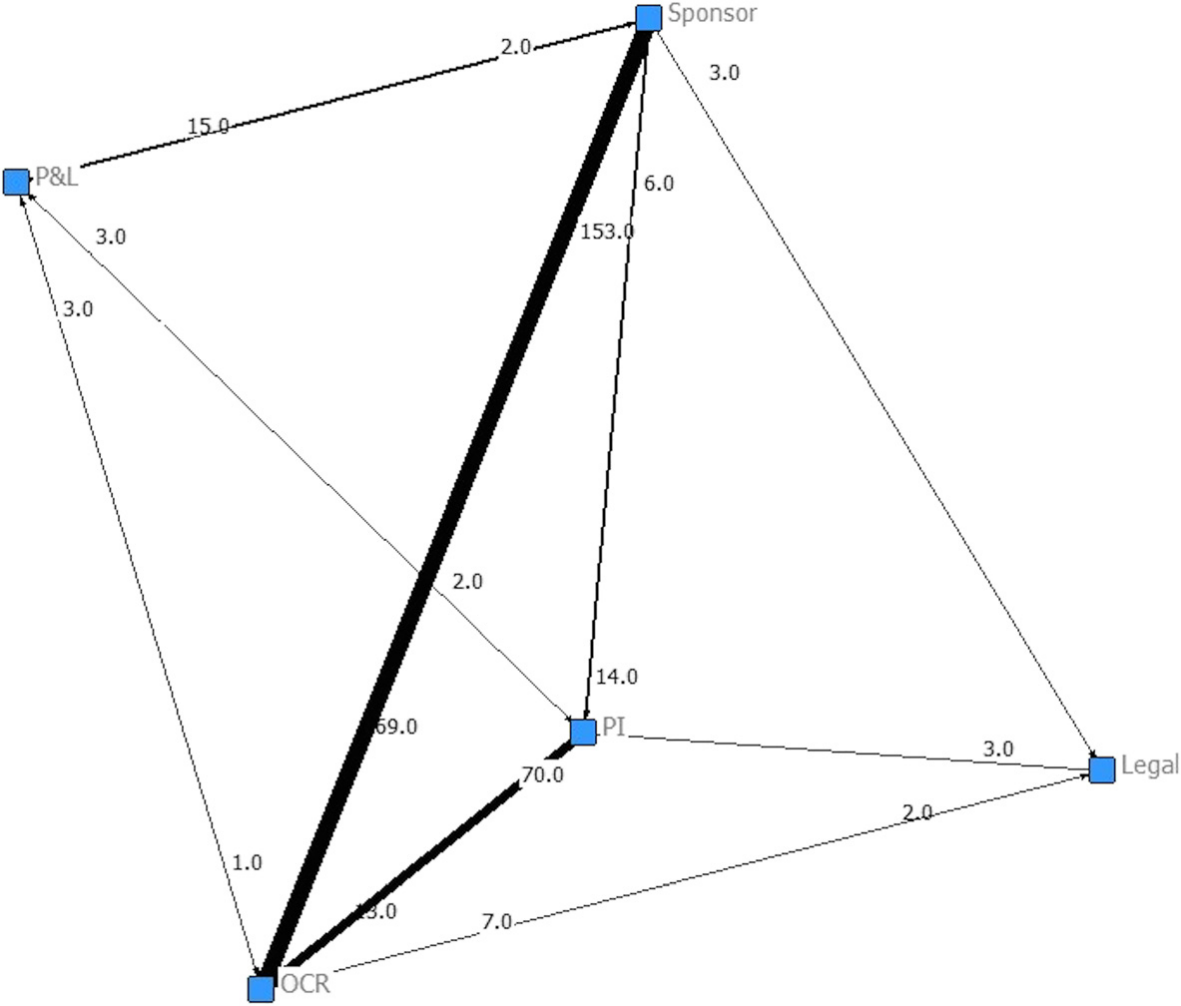
Social network diagram for Budget Negotiation. Each node represents a participant; a tie denotes an interaction between participants; tie width denotes the frequency of interactions between two participants. The number closest to the node represents the number of interactions started in the node to others. During Budget Negotiation the majority of interactions occur between OCR, sponsor, and PI. *DSR* USF’s Division of Sponsored Research, *OCR* USF’s Office of Clinical Research, *P&L* USF’s Division of Patents and Licensing, *PI* principal investigator

During Contract Negotiation (Fig. 2) the majority of interactions occur between OCR and sponsors. OCR initiates more interactions than the sponsor (507 versus 346), and has the highest in- and out-degree (see Table 3). These results highlight OCR’s active role during contract development as well as suggesting a higher degree of complexity during contract development activities than those of budgeting. During Budget Negotiation (Fig. 3) the majority of interactions occur between OCR, sponsors, and PIs. Surprisingly, sponsor and PI initiate more interactions than OCR (153 versus 69 and 70 versus 13, respectively) suggesting that OCR has been effective in stimulating PIs and sponsors in being more responsive or that OCR has been inefficient in responding to PIs and sponsors requests. Post-hoc analyses demonstrated that OCR response time during Contract Negotiation and Budget Negotiation are significantly lower than the response time of the PIs and sponsors. Any process improvement strategy should be focused on these three participants as well as aiming at stimulating PIs and sponsors in being more responsive to OCR.

**Table 3:** Centrality measures for Contract Negotiation and Budget Negotiation

Sub-process	Participant	Degree		Betweenness	Closeness		Bonacich’s Power Index
		In	Out		In	Out	
Contract Negotiation	OCR	6.0	6.0	1.5	5.0	5.0	1.9
	Sponsor	5.0	5.0	1.5	5.0	5.0	1.5
	PI	4.0	3.0	0.0	4.5	4.0	0.2
	P&L	3.0	5.0	0.0	4.0	5.0	0.2
	USF Legal	4.0	5.0	0.7	4.5	5.0	0.5
	DSR	5.0	3.0	0.3	5.0	4.0	0.1
Budget Negotiation	OCR	5.0	5.0	2.0	4.0	4.0	1.2
	Sponsor	3.0	4.0	1.0	3.5	4.0	1.7
	PI	4.0	3.0	1.0	4.0	3.5	0.9
	P&L	3.0	3.0	0.0	3.5	3.5	0.3
	Legal	2.0	3.0	0.0	3.0	3.0	0.1

*DSR* USF’s Division of Sponsored Research, *OCR* USF’s Office of Clinical Research, *P&L* USF’s Division of Patents and Licensing, *PI* principal investigator

### Simulation model

A detailed description of the simulation model can be found in Additional file 2: Figure SI1. We validated the simulation model, as shown in Table 4, by comparing actual versus simulated system performance measures. At baseline we assume that 14 trials per month on average are received at the OCR. The largest divergence was found in the number of trials still in process (52 versus 78.6 days). Increasing the number of replications shows no significant effect on this divergence, which is likely caused by offsets in the model. This can be improved by considering collecting data from those trials that are still in process. However, the model can still support decision-making by providing information on delays, durations, queue lengths, and resource utilizations. It is important to note that the clinical study site is in charge of either WIRB or IRB submission, not OCR. Therefore, detailed activities related to WIRB Approval and IRB Approval are not included in the simulation model.

**Table 4:** Comparison of actual versus simulated system performance measures. Simulated data was produced by 38 replications of the simulation model

Sub-process	Average duration in calendar days	
	Actual	Simulated (95 % CI)
Initial Preparation	11.0^a^	10.9 (0.1)
Contract Negotiation	54.9	51.6 (4.5)
Budget Negotiation	46.3	45.3 (3.9)
PI Approval	4.8	4.6 (0.2)
DSR Approval	2.5	2.4 (0.1)
Sponsor Approval	5.5	5.2 (0.4)
Entire Process	76.6	82.0 (3.5)
Trials arriving/leaving	Average number per year	
	Actual	Simulated (95 % CI)
Number in	147.0	143.7 (4.3)
Number out	78.0	74.5 (3.2)
Still in process	52.0	78.6 (5.1)

*CI* confidence interval, *DSR* USF’s Division of Sponsored Research, *PI* principal investigator

^a^Estimated by OCR senior personnel

To demonstrate how the model can assist in detecting an effective process-improvement scenario, we conducted 5 different numerical studies with the OCR as a sample system. Note that one can test any combination of these scenarios as demonstrated in the following experiments.

#### Analysis of system capacity

Between 2009 and 2013, the total number of clinical trials registered in ClinicalTrials.gov has increased by 91 % (from 83,467 to 157,371). Although in recent years there has been a shift in industry-sponsored trials moving to emerging countries in Eastern Europe, South America, and Asia; still the United States dominates by a large margin having more than eight times more clinical trial sites than the country in second place, Germany [34]. To study the tolerance of the process in terms of the uncertainty in the number of trials seeking activation we conducted the following analysis. If the expected number of trials arriving to OCR deviates from expectations, what will be the effect on the activation time? We gradually increased the average arrival rate from 14 to 24 trials per month. Our simulation results indicate that even a slight increase would cause significant delays to trial activation (see Additional file 2: Figure SI3). For instance, an increase from 14 to 16 trials per month would increase the activation time by 11 % (from 82 to 90.7 days, 95 % confidence). Similar effect is noted in the total idle time increasing by 24 % (from 40.1 to 49.8 days, 95 % confidence) (see Additional file 2: Figure SI3). The queue lengths for Contract Negotiation and Budget Negotiation would also increase by 41 % (from 18.5 to 26.1 trials, 95 % confidence) and by 43 % (from 15.2 to 21.7 trials, 95 % confidence), respectively (see Additional file 2: Figure SI3b).

#### Analysis of key participants’ capacity

**Fig. 4:**
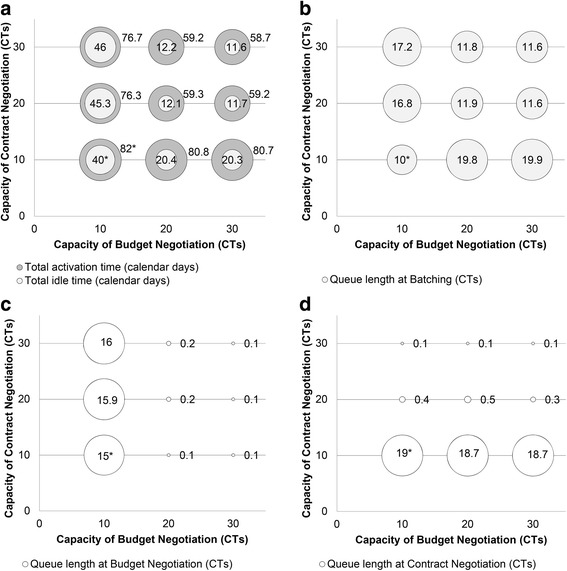
How additional resource capacity in Contract Negotiation and Budget Negotiation can affect system performance. Additional capacity will reduce: **a** Average activation time. **a** Idle time. **b**, **c** and **d** Queue length. *Baseline scenario

Contract Negotiation and Budget Negotiation have been identified as the process performance drivers. To optimize resource allocation, it is necessary to identify which sub-process is causing the largest deviation in total activation time. Here we examine the system’s sensitivity to changes on key participants’ capacity, i.e., the number of trials that a participant can handle at a given time that can be changed based on effort allocation. It is important to note that improving the capacity for Contract Negotiation and Budget Negotiation will require join efforts from OCR, sponsors, and PIs, due to the existence of reciprocal activities during contract and budget development. As shown in Fig. 4a, our simulation results indicate that doubling the capacity for both Contract Negotiation and Budget Negotiation may significantly improve system performance. Average activation time would be reduced by 28 % (from 82 to 59.3 days, 95 % confidence) and average idle time would be reduced by 70 % (from 40.1 to 12.1 days, 95 % confidence) (Fig. 4a). Along with activation time reductions, the average number of trials in queue for Contract Negotiation and Budget Negotiation would be reduced to almost zero (95 % confidence) (Fig. 4b, c, and d). Important to note in Fig. 4a is that additional increases of capacity may fail to further improve system performance, as does incrementing capacity for just one of the key participants.

#### Analysis of key participants’ capacity under increased workload

A critical assumption in the previous analyses is that the average number of trials arriving to OCR remains at 14 trials per month on average. To test the robustness of our previous results, it is necessary to identify process performance drivers under increased workload. To simulate the increase workload scenario, we doubled the average arrival rate from 14 to 28 trials per month. Such workload increases total completion time by 40 % (from 82 to 114.6 days, 95 % confidence) and idle time by 110 % (from 40.1 to 84 days, 95 % confidence) (see Additional file 2: Figure SI4a). Our simulation results indicate that doubling the capacity for both, Contract Negotiation and Budget Negotiation, would be necessary to keep system performance near to its baseline performance of 82 days’ average activation time. Adding more capacity for both sub-processes would result in further improvements by reducing average completion time by 45 % (from 114.6 to 63.2 days, 95 % confidence) and idle time by 83 % (from 84 to 14.4 days, 95 % confidence). Important to note is that unbalanced addition of capacity (i.e., tripling capacity for Contract Negotiation and keeping Budget Negotiation as it is, or vice versa) would result in no improvements. Adding capacity will reduce queue lengths for Contract Negotiation and Budget Negotiation (see Additional file 2: Figures SI4c and SI4d). However, queue length for batching will increase as personnel increase (Additional file 2: Figure SI4b). This is because Contract Negotiation and Budget Negotiation sub-processes have different completion times. Therefore, more trials must wait for either budget or contract to finish before moving to the next sub-process.

#### Analysis of other participants’ capacity

Relaxing the conditions at which sub-processes other than Contract Negotiation and Budget Negotiation are performed represents an opportunity to save resources. In this scenario, we assume that sub-processes outside OCR are a cause of trial activation delay. Even though OCR cannot control the performance of these sub-processes, it can utilize automated reminder systems in hopes that it will reduce their response time. It is expected that the performance of the entire process will not change because the rate-limiting sub-processes, Contract Negotiation and Budget Negotiation, are not modified. As expected, our simulation results indicate that increasing the capacity of sub-processes other than Contract Negotiation and Budget Negotiation (see Additional file 2: Figure SI5) would not have a statistical significant (95 % confidence) effect on reducing average activation time, idle time, or queue length. Our findings confirm the need to focus process-improvement efforts on contract and budget development activities.

#### Analysis of processing time variability

**Fig. 5:**
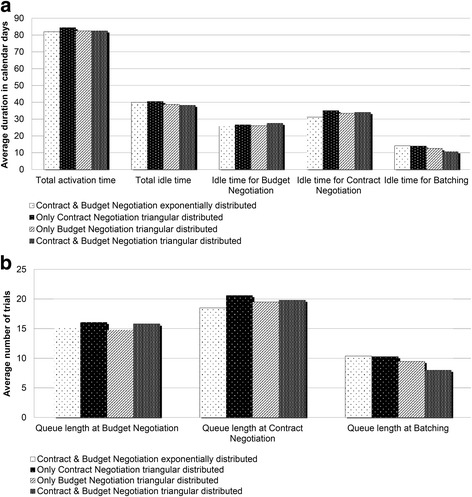
Reducing processing time variability in Contract Negotiation and Budget Negotiation would result in significant process improvements

The previous analyses show that contract development and budgeting are the performance drivers of the process. Regrettably, historical timing data show that both have a high variability (34.6 and 50.3 standard deviation, respectively), which is attributed to the natural complexity of the process and a lack of coordination between personnel involved with Contract Negotiation and Budget Negotiation. In this experiment, we evaluate the system’s performance if the variability in both sub-processes is reduced. We hypothesize that a better communication and synchronization should reduce such variability. We model reduction in processing variability by replacing exponentially distributed processing times with triangular distributed processing times. Triangular distribution is commonly used when estimates for the minimum, maximum, and most likely values are known [35]. As shown in Fig. 5a, the system performance is significantly improved in terms of time spent for each trial at batching (from 14.2 to 10.8 days, 95 % confidence). Such reduction was expected because of the simulated improvement on synchronization between Contract Negotiation and Budget Negotiation. Nonetheless, these improvements do not translate into further reductions of activation time (Fig.5a) or queue lengths (Fig. 5b)

## Discussion

This study proposes a comprehensive framework to aid process improvement decision-making, which was used to assess the opening of industry-sponsored clinical trials at the USF’s Office of Clinical Research. Although several attempts have been made to examine the operational efficiency of trial activation [6, 8–10, 13], most of the previous work concentrates solely on a certain aspect of improvement such as barrier identification or process reconfiguration. More recent studies have shown improvements using six sigma and lean manufacturing tools (e.g., [14, 17, 18, 22]); however, their mass approach might not be suitable across all academic medical settings or might not go well with other organizational cultures. We propose a framework based on a totally different approach that maps the administrative process through VSM, identifies barriers for trial opening through timing analysis and SNA, and examines system performance under different scenarios through DES. The use of systematic approaches to analyze the trial activation process, rather than case study alone, has been recognized as a key tool to make trials more efficient [21].

We found that the mean time to activate an industry-sponsored clinical trial at USF is 76.6 days (median = 69 days). The administrative process comprises 5 sub-processes, 30 activities, 11 decision points, 5 loops, and 8 participants. Contract Negotiation and Budget Negotiation are the most complex and time-consuming sections of the administrative process, lasting on average 54.9 and 46.3 days, respectively. This finding is in accordance with the work reported elsewhere [8, 9]. It is surprising, however, that the longest sub-process is not IRB Approval as believed by the research community.

Using SNA, we found that within Contract Negotiation and Budget Negotiation the OCR, sponsor, and PI are the most central participants. This finding was incorporated into the process improvement scenarios tested in the DES model. For instance, we found that by increasing the capacity on contract and budgeting development at OCR the mean activation time may be reduced by 28 %, mean idle time by 70 %, and queue length to almost zero. However, additional increase may not result into further improvements. To demonstrate the extensive capabilities of our approach we successfully simulated and analyzed four more strategies. Besides the scenarios reported in this study, there are other strategies that can be used to reduce activation time and can be tested using our framework before actual implementation. For instance, the use of Master Agreements and Previously Negotiated Terms would make the administrative process nimble by reducing the time spent on Contract Negotiation and Budget Negotiation; the parallelization of activities within the administrative process is likely to generate efficiency improvements as shown elsewhere (e.g., [18, 36, 37]); and, the standardization of contracts across industry, academia, and federal agencies may reduce prolonged delays in obtaining regulatory approvals to initiate patient enrollment [38].

Our research has limitations. First, the presented framework relies heavily on data that are not routinely collected. Second, validation of the proposed scenarios has only been achieved through simulation and, therefore, the results may not be attainable in real settings, where human factor analysis and change management may be required. Third, duration estimations may represent a lower bound of the actual durations, due to the fact that trials still in process were excluded. Fourth, industry communication between the PI and the sponsor was not considered into the SNA. Future research should focus on studying the interactions between PIs and sponsors, which are likely to provide meaningful insights to devise improvement scenarios. Finally, we have assessed the system performance in terms of improvements in activation time, idle time, and queue lengths, and not in terms of health outcomes. This is a very critical observation and its effects are currently overwhelming health research administration. Improvements in process design do not necessarily translate to improvements in health outcomes. Additional research is required to address this limitation. Nevertheless, we believe that in the hands of clinical research administrators, the proposed framework is useful to assess the process of activating trials.

## Conclusions

We present a novel approach to understanding drivers of process performance during the opening of clinical trials. Specifically, we analyzed the current state of the process using VSM, described the interactions of the various process participants using SNA, and evaluated the potential impact of process modifications using DES. We advance previous research by incorporating SNA to better understand the roles and interactions of the various participating entities, as well as DES to model potential modifications and scenarios of increased workload. In the hands of clinical research administrators, our approach holds promise for improving efficiency and supporting better-informed prioritization and resource allocation. Overcoming administrative barriers to opening clinical trials may result in augmenting patient treatment options without compromising research integrity or patient safety. Deploying systems engineering tools provide the ability to leverage naturally generated administrative information to perform more evidence-based management of clinical trials.

## Additional files

Additional file 1:
**Process duration data for the trials included in the study**. (XLSX 83 kb) Additional file 2: Figure SI1. Simulation logic for the administrative process associated with industry-sponsored clinical trial activation. Abbreviations: CDA/NDA, confidential disclosure agreement or non-disclosure agreement; CT, clinical trial; DSR, USF’s Division of Sponsored Research; PI, principal investigator. Figure SI2. Administrative procedure involved in the opening of clinical trials managed by the Office of Clinical Research at the University of South Florida. At the level of granularity, the process for activating an industry-sponsored clinical trial at USF comprises 30 steps, 11 decisions, 4 loops, and 8 participants. Abbreviations: CDA/NDA, confidential disclosure agreement or non-disclosure agreement; CTA, clinical trial agreement; OCR, USF’s Office of Clinical Research; P&L, USF’s Patents and Licensing; PI, principal investigator; SRA, OCR’s Senior Research Assistant; WIRB, Western Institutional Review Board. Figure SI3. Analysis of system capacity. Increasing the number of clinical trials arriving at the Office of Clinical Research. Slight increase will cause statistically significant delays to clinical trial activation, increase idle time, and queue lengths. Abbreviations: CTs, clinical trials. Figure SI4. Analysis of key participants’ capacity under increased workload. Effect of personnel addition under an increased demand scenario of 28 clinical trials per month average. At least two new employees must be assigned to OCR in order to maintain the baseline system performance. Abbreviations: CTs, clinical trials. *Baseline scenario. Figure SI5. Analysis of other participants’ capacity. Reducing response time from participants outside USF’s Office of Clinical Research. Reducing response time would not have a statistically significant (95 % confidence) effect on average activation time, idle time, and queue length. Figure SI6. *T* test comparing the mean duration of Contract Negotiation with and without data of those clinical trials still in process. Figure SI7. *T* test comparing the mean duration of Budget Negotiation with and without data of those clinical trials still in process. (DOCX 3073 kb)

## Abbreviations

DES: discrete-event simulation
DSR: USF’s Division of Sponsored Research
IRB: Institutional Review Board
OCR: Office of Clinical Research
PI: principal investigator
USF: University of South Florida
VSM: value stream mapping
SNA: social network analysis
WIRB: Western Institutional Review Board
