# Supplementary material for: Activating clinical trials: a process improvement approach
Source: Trials. 2016 Feb 24;17:106. doi: 10.1186/s13063-016-1227-2 (PMC4765218; doi:10.1186/s13063-016-1227-2)
Supplement: Additional file 2: Figure SI1. — Simulation logic for the administrative process associated with industry-sponsored clinical trial activation. Abbreviations: CDA/NDA, confidential disclosure agreement or non-disclosure agreement; CT, clinical trial; DSR, USF’s Division of Sponsored Research; PI, principal investigator. Figure SI2. Administrative procedure involved in the opening of clinical trials managed by the Office of Clinical Research at the University of South Florida. At the level of granularity, the process for activating an industry-sponsored clinical trial at USF comprises 30 steps, 11 decisions, 4 loops, and 8 participants. Abbreviations: CDA/NDA, confidential disclosure agreement or non-disclosure agreement; CTA, clinical trial agreement; OCR, USF’s Office of Clinical Research; P&L, USF’s Patents and Licensing; PI, principal investigator; SRA, OCR’s Senior Research Assistant; WIRB, Western Institutional Review Board. Figure SI3. Analysis of system capacity. Increasing the number of clinical trials arriving at the Office of Clinical Research. Slight increase will cause statistically significant delays to clinical trial activation, increase idle time, and queue lengths. Abbreviations: CTs, clinical trials. Figure SI4. Analysis of key participants’ capacity under increased workload. Effect of personnel addition under an increased demand scenario of 28 clinical trials per month average. At least two new employees must be assigned to OCR in order to maintain the baseline system performance. Abbreviations: CTs, clinical trials. *Baseline scenario. Figure SI5. Analysis of other participants’ capacity. Reducing response time from participants outside USF’s Office of Clinical Research. Reducing response time would not have a statistically significant (95 % confidence) effect on average activation time, idle time, and queue length. Figure SI6. T test comparing the mean duration of Contract Negotiation with and without data of those clinical trials still in process. Figure SI7. T test comparing th [file 13063_2016_1227_MOESM2_ESM.docx]

**ACTIVATING CLINICAL TRIALS: A PROCESS IMPROVEMENT APPROACH**

**METHODS**

**Discrete Event Simulation Model**

The logic of the simulation model is described in Figure SI1. Each box represents a sub-process to be completed before a trial is activated. The Batch Budget and Contract box is used to model the delay imposed due to synchronization issues between completion of Contract Negotiation and Budget Negotiation. Trials enter the simulation randomly. Events occur based on time distributions described in Table 1. Once a trial enters the system it is immediately routed to Initial Preparation, and then it is routed to Contract Negotiation and Budget Negotiation. Upon completion, budget and contract are merged and routed to the last three sub-processes called PI Approval, DSR Approval, and Sponsor Approval. Once OCR receives these three approvals, the clinical trial is considered activated and ready for patient accrual.


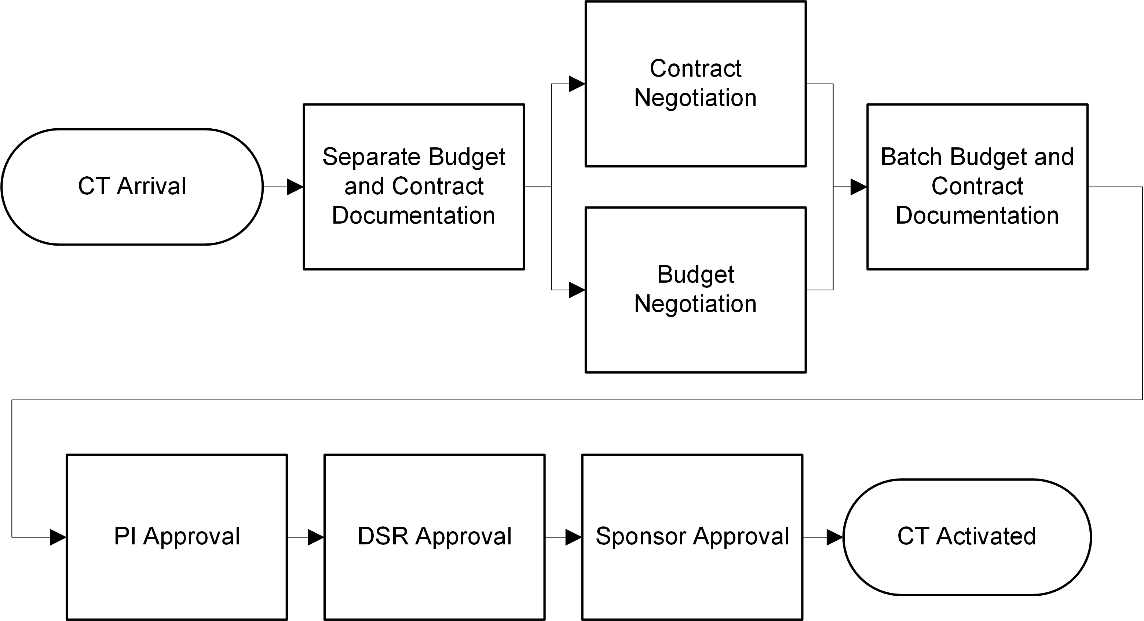


**Figure SI1**. Simulation logic for the administrative process associated with industry-sponsored clinical trial activation. Abbreviations: CDA/NDA, confidential disclosure agreement or non-disclosure agreement; CT, clinical trial; PI, principal investigator; DSR, USF’s Division of Sponsored Research.

**RESULTS**

**Process mapping and timing analysis**

The administrative process associated with trial activation is depicted in Figure SI2. Five major sub-processes can be recognizable in the process: Initiation; Budget Negotiation; Contract Negotiation; WIRB (or IRB) Preparation; and, Finalization.

During *Initiation* OCR receives the legal contract (i.e., confidentiality disclosure agreement - CDA) from sponsors, which is then routed to the USF’s Division of Patents and Licensing (P&L) for its revision and execution. The executed CDA is distributed to sponsors, PI, and OCR. The PI submits the research protocol and consent forms for revision to OCR. Upon reception, the following three sub-processes begin simultaneously: Contract Negotiation, Budget Negotiation, and WIRB Preparation (if needed). During *Contract Negotiation*, OCR reviews the contract language and consent forms in accordance with institutional requirements. During Budget Negotiation, OCR facilitates budget negotiations and ensures that payment terms meet necessary institutional requirements. During *WIRB Preparation*, OCR provides assistance to prepare the necessary documentation to go through commercial IRB (e.g., WIRB) revision. It is important to note is that the preparation for commercial IRB submission is different than the actual IRB revision process. In addition, OCR only provides assistance for commercial IRB submissions and not for USF IRB submissions. There exists an independent unit at USF that is responsible for assisting researchers that need to go through USF IRB revision. Finally, during the *Finalization* sub-process the final revisions and approval signatures are collected from each participant. This is the end point of the administrative process, and the trial is considered ready for patient accrual.

As detailed in Table SI1, the administrative process at OCR comprises 30 activities, 11 decision points, 4 loops, and 8 participants. Contract Negotiation corresponds to the most complex sub-process, since it has the highest number of participants, activities, and loops. Most frequent non-value-added activities identified throughout the entire process include: waiting for merging Contract and Budget documentation after they have been agreed upon; obtaining pending documentation; uploading documentation to a shared data management system; and obtaining final approval signatures. The proportion of non-value-added activities in each sub-process ranges from 29 to 80%, with Budget Negotiation demonstrating the highest value.

**Table SI1**. Number of participants, activities, loops, and decision points in the administrative process associated with industry-sponsored clinical trial activation at the University of South Florida. Value defined as those activities that impact quality, safety, or effectiveness of the clinical trial. Abbreviations: WIRB, Western Institutional Review Board; VA, value-added activities; NVA, non-value-added activities.

| **Sub-process** | **Participants** | **VA** | **NVA (%)** | **Loops** | **Decision Points** |
| --- | --- | --- | --- | --- | --- |
| Initial Preparation | 4 | 3 | 4 (57) | 2 | 3 |
| Contract Negotiation | 5 | 5 | 3 (37) | 1 | 2 |
| Budget Negotiation | 3 | 1 | 4 (80) | 1 | 3 |
| Final Negotiation | 4 | 1 | 2 (77) | 0 | 0 |
| WIRB Preparation | 4 | 5 | 2 (29) | 0 | 3 |
| Entire Process | 8 | 15 | 15 (50) | 4 | 11 |


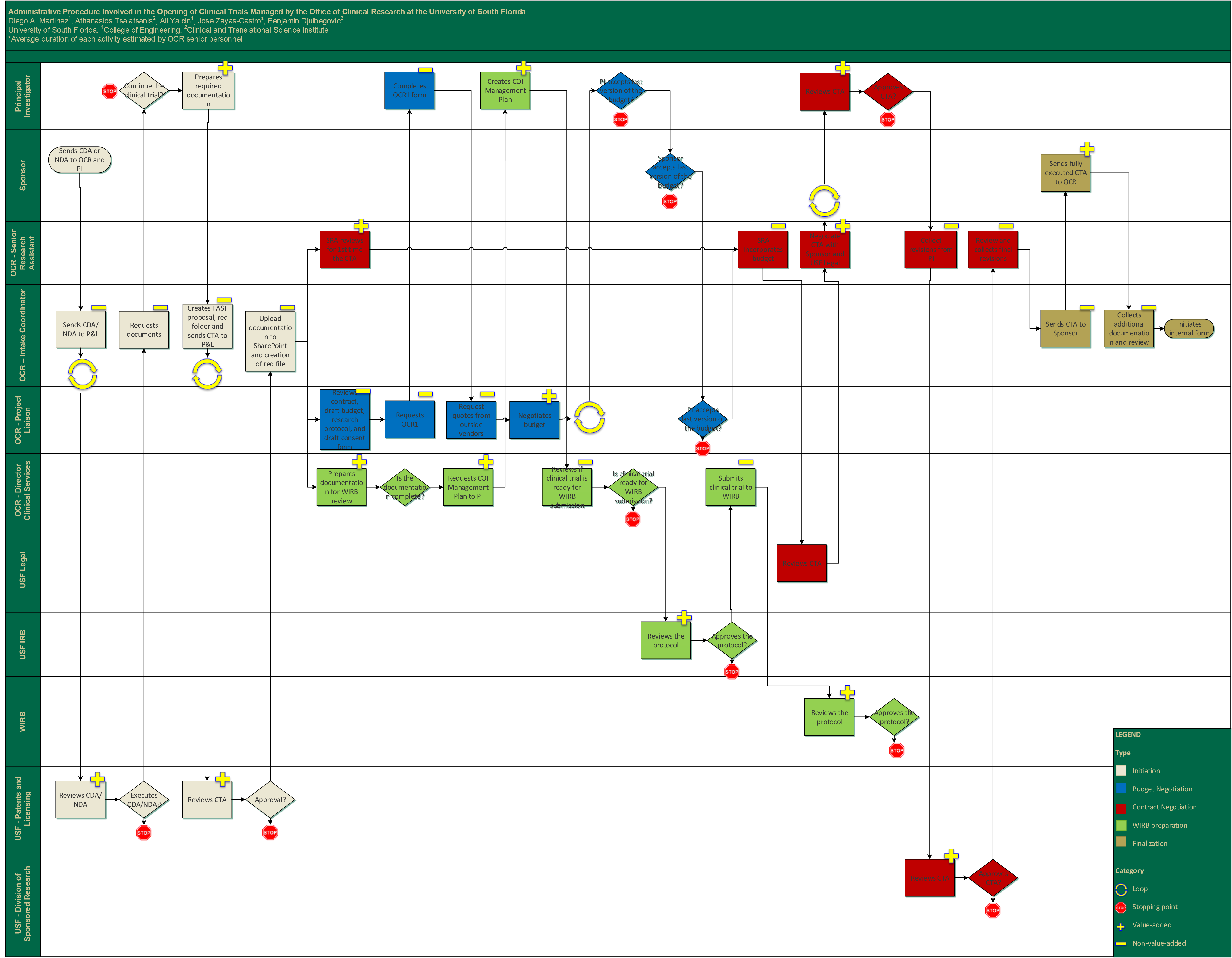


**Figure SI2**. Administrative procedure involved in the opening of clinical trials managed by the Office of Clinical Research at the University of South Florida. At the level of granularity, the process for activating an industry-sponsored clinical trial at USF comprises 30 steps, 11 decisions, 4 loops, and 8 participants. Abbreviations: CDA/NDA, confidential disclosure agreement or non-disclosure agreement; PI, principal investigator; P&L, USF’s Patents and Licensing; OCR, USF’s Office of Clinical Research; SRA, OCR’s Senior Research Assistant; WIRB, Western Institutional Review Board; CTA, clinical trial agreement.

**Simulation Results**

**Analysis of system capacity.** It is expected that the number of trials arriving to the USF’s Office of Clinical Research (OCR) will only increase in the following years. Based on this expectation, we conducted the following experiment using the simulation model. If the expected number of clinical trials seeking for activation through OCR increases, what will be the effect on the clinical trial activation time? We gradually increase the average arrival rate from 14 to 24 trials per month. As presented in Figure SI3a, slight increments in the average demand would cause significant delays to trial activation. For instance, an increase from 14 to 16 trials per month would increase the activation time by 11% (from 82 to 90.7 days, 95% confidence). Similar effect is noted in total idle time increasing by 24% (from 40.1 to 49.8 days, 95% confidence) and in queue lengths for Contract Negotiation and Budget Negotiation, which would also increase by 41% (from 18.5 to 26.1 trials, 95% confidence) and by 43% (from 15.2 to 21.7 trials, 95% confidence), respectively (see Figure SI3b).


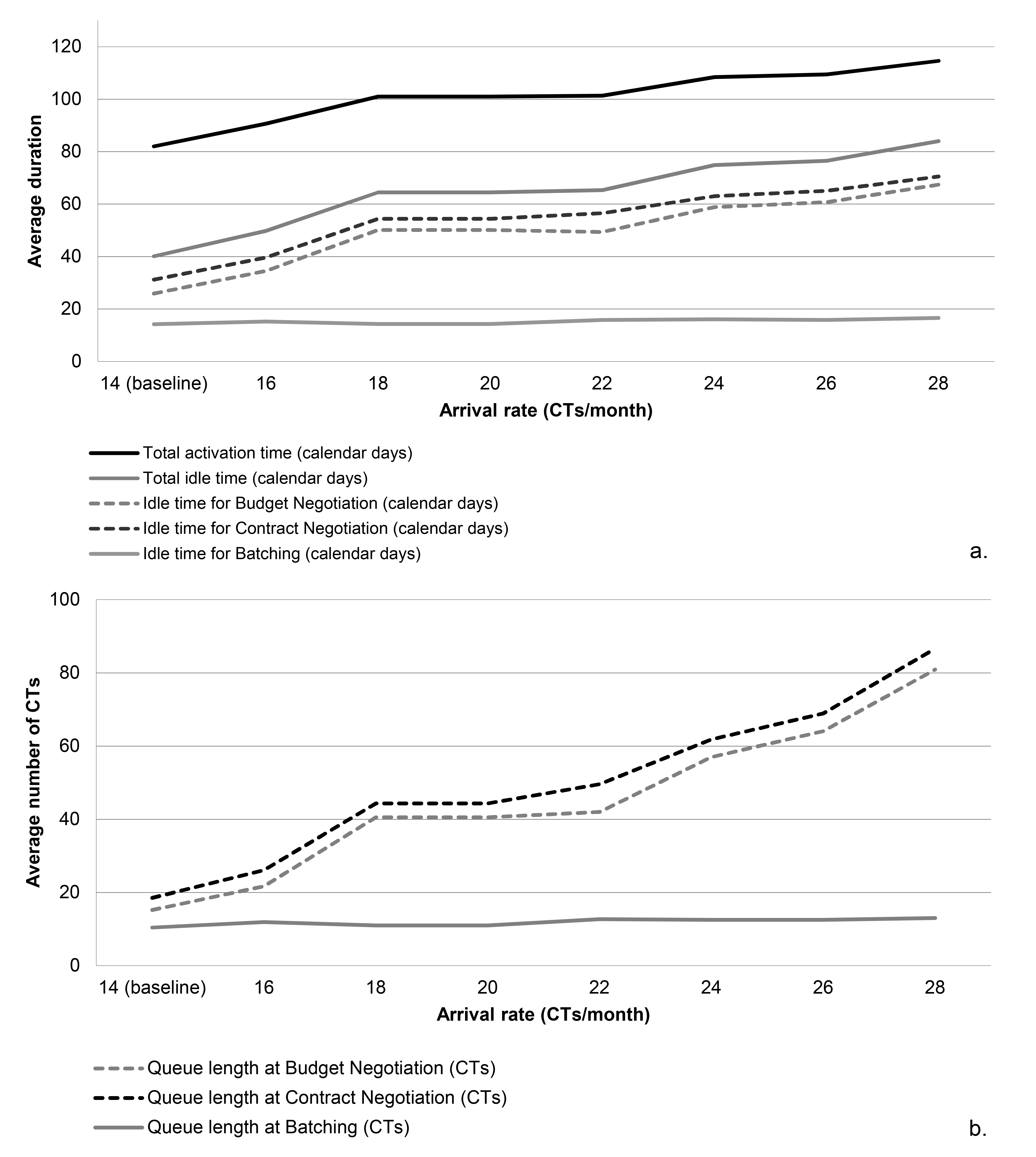


**Figure SI3**. Analysis of system capacity. Increasing the number of clinical trials arriving at the Office of Clinical Research. Slight increase will cause statistically significant delays to clinical trial activation, increase idle time, and queue lengths. Abbreviations: CTs, clinical trials.

**Analysis of key participants’ capacity under increased workload.** A critical assumption in the previous analysis is that the average number of trials arriving to OCR remains constant at 14 trials per month. To test the robustness of our previous results, it is necessary to identify process performance drivers under increased workload. We doubled the average arrival rate from 14 to 28 trials per month, and then analyzed the impact on process performance. As presented in Figure SI4a, the total completion time increased by 40% (from 82 to 114.6 days, 95% confidence) and the idle time by 110% (from 40.1 to 84 days, 95% confidence). We then artificially increased the capacity for both Contract Negotiation and Budget Negotiation finding that, doubling the capacity for both sub-processes is necessary to keep the system performance near to its baseline performance of 82-days activation time. Adding more capacity for both sub-processes would result in further improvements by reducing average completion time by 45% (from 114.6 to 63.2 days, 95% confidence) and idle time by 83% (from 84 to 14.4 days, 95% confidence). Important to note is that unbalanced addition of capacity (e.g., tripling capacity for Contract Negotiation and keeping Budget Negotiation as it is, or vice versa) would result in no improvements. Adding capacity will reduce queue lengths for Contract Negotiation and Budget Negotiation (see Figures SI4c and SI4d). However, queue length for batching will increase as personnel increases (Figure SI4b). This is because Contract Negotiation and Budget Negotiation sub-processes have different completion times. Therefore, more trials must wait for either budget or contract to finish before moving to the next sub-process.


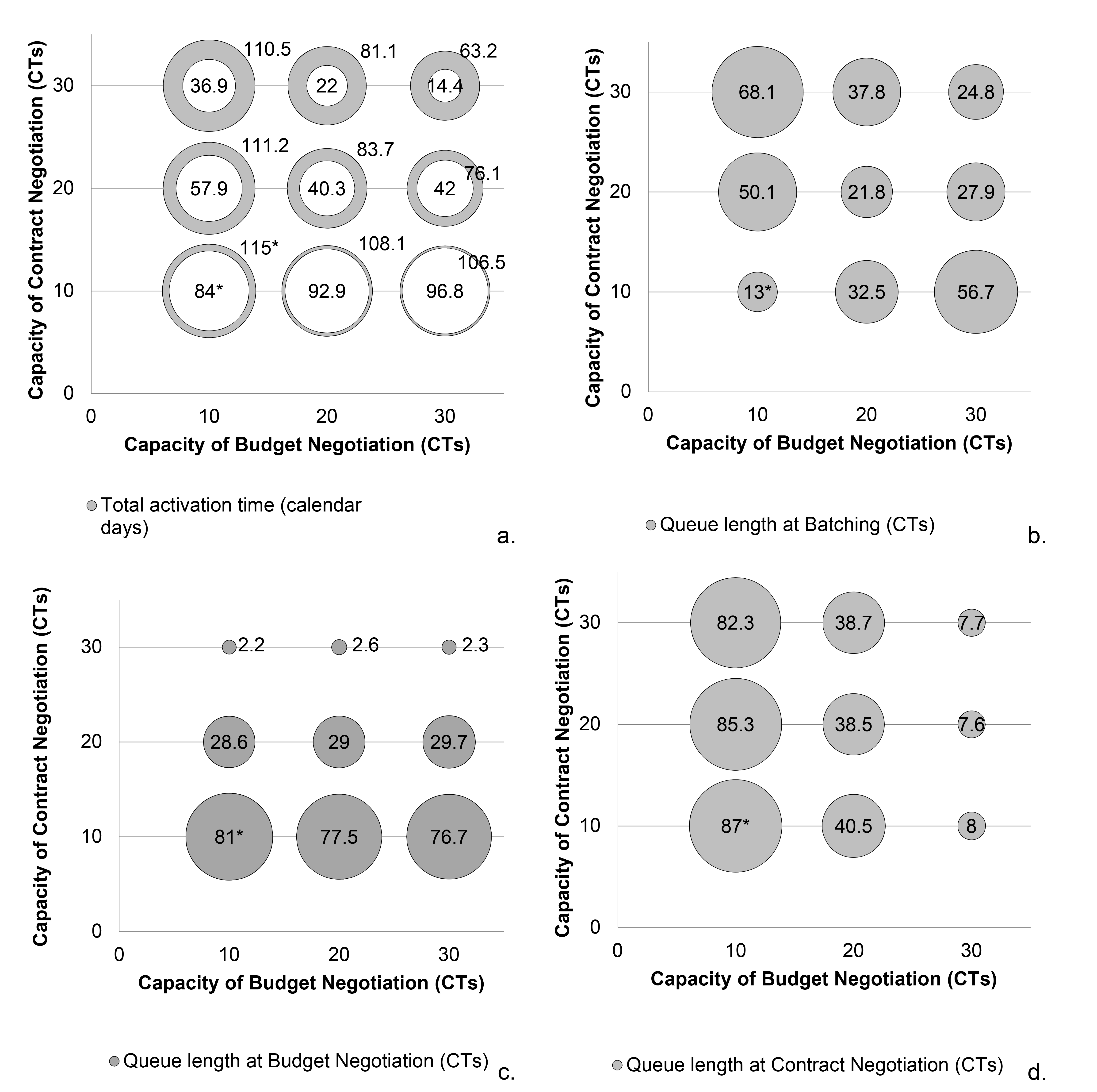


**Figure SI4**. Analysis of key participants’ capacity under increased workload. Effect of personnel addition under an increased demand scenario of 28 clinical trials per month average. At least two new employees must be assigned to OCR in order to maintain the baseline system performance. Abbreviations: CTs, clinical trials. *Baseline scenario.

### Analysis of other participants’ capacity. Relaxing the performance at which sub-processes other than Contract and Budget Negotiation are performed represents an opportunity to save resources. These resources can be used on those rate-limiting steps to improve overall process efficiency. In this scenario, we assume that sub-processes outside OCR are a cause of trial activation delay. Even though OCR cannot control the performance of these sub-processes, it can utilize automated reminder systems in hopes that it will reduce their response time. It is expected that the performance of the entire process will not change because the rate-limiting sub-processes, Contract and Budget Negotiation, are not modified. As expected, our simulation results indicate that increasing the capacity of sub-processes other than Contract and Budget Negotiation (see Figure SI5) would not have a statistical significant (95% confidence) effect on reducing average activation time, idle time, or queue length. Suggesting that resources spent on those sub-process can be used to accelerate the contract and budget development activities without affecting activation time.


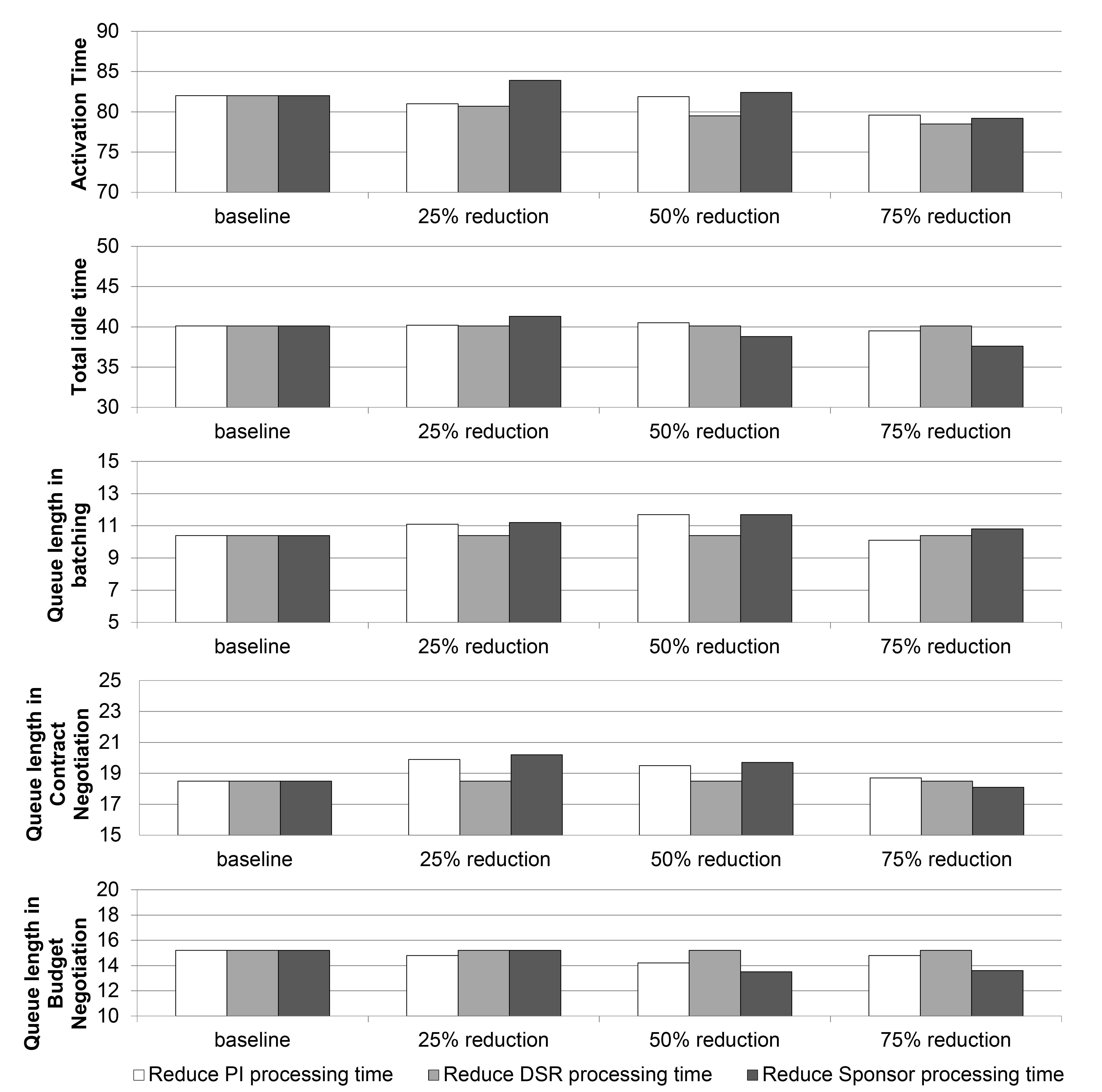


**Figure SI5**. Analysis of other participants’ capacity. Reducing response time from participants outside USF’s Office of Clinical Research. Reducing response time would not have a statistically significant (95% confidence) effect on average activation time, idle time, and queue length.

**Post-hoc analysis for exclusion of clinical trials still in process.** The exclusion of clinical trials still in process when calculating the processing times of Contact Negotiation and Budget Negotiation may form the basis for a significant bias, as recognized in the limitations of our paper. As shown in Figures SI6 and SI7, we found no significant differences in the durations of Contract Negotiation (54.91 versus 57.95, P-Value=0.6993) and Budget Negotiation (46.3 versus 60.76, P-Value=0.0712) after adding duration data of the clinical trials still in process. Therefore, we conclude that the exclusion of the trials still in process formed no statistically significant bias. A detailed description of our statistical analysis is presented next.

- **Comparison of the average duration of Contract Negotiation for completed clinical trials versus still in process clinical trials.**

We must first check whether the variances are homogeneous (homoskedasticity) with a F-test of Fisher:

F test to compare two variances

data: Contract_WithStillInProcess and Contract_WithoutStillInProcess

F = 0.91597, num df = 90, denom df = 68, p-value = 0.6919

alternative hypothesis: true ratio of variances is not equal to 1

95 percent confidence interval:

0.5806754 1.4245380

sample estimates:

ratio of variances

0.9159719

Since the obtained p-value is greater than 0.05, we failed to reject the null hypothesis of homogeneity of variances. We therefore use independent two-sample t-test for samples with equal variance:

Two Sample t-test

data: Contract_WithStillInProcess and Contract_WithoutStillInProcess

t = 0.38691, df = 158, p-value = 0.6993

alternative hypothesis: true difference in means is not equal to 0

95 percent confidence interval:

-12.44580 18.50982

sample estimates:

mean of x mean of y

57.94505 54.91304

Since the obtained p-value is greater than 0.05, we failed to reject the null hypothesis that the means of the populations from which the two samples were taken are equal.

- **Comparison of the average duration of Budget Negotiation for completed clinical trials versus still in process clinical trials.**

We must first check whether the variances are homogeneous (homoskedasticity) with a F-test of Fisher:

F test to compare two variances

data: Budget_WithStillInProcess and Budget_WithoutStillInProcess

F = 1.6033, num df = 79, denom df = 39, p-value = 0.106

alternative hypothesis: true ratio of variances is not equal to 1

95 percent confidence interval:

0.9025037 2.7054070

sample estimates:

ratio of variances

1.603339

Since the obtained p-value is greater than 0.05, we failed to reject the null hypothesis of homogeneity of variances. We therefore use independent two-sample t-test for samples with equal variance

Two Sample t-test

data: Budget_WithStillInProcess and Budget_WithoutStillInProcess

t = 1.8206, df = 118, p-value = 0.0712

alternative hypothesis: true difference in means is not equal to 0

95 percent confidence interval:

-1.268457 30.193457

sample estimates:

mean of x mean of y

60.7625 46.3000

Since the obtained p-value is greater than 0.05, we failed to reject the null hypothesis that the means of the populations from which the two samples were taken are equal.


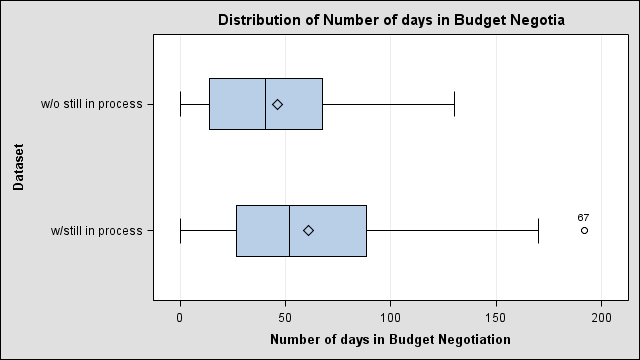


**Figure SI6.** Distribution of the number of days each clinical trial spent in Contract Negotiation. In the control group, no significant change occurred in the duration of Contract Negotiation from baseline to w/still in process clinical trials added. The t-test was used to compare differences between groups.


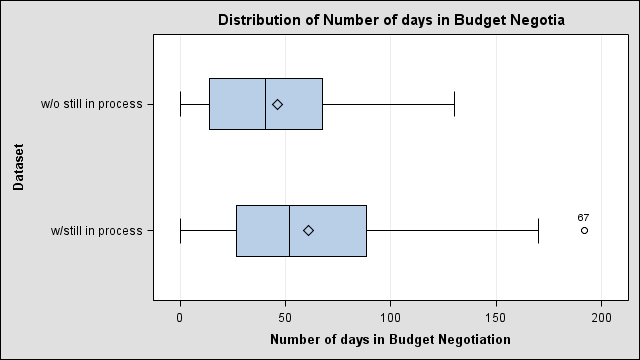


**Figure SI7.** Distribution of the number of days each clinical trial spent in Budget Negotiation. In the control group, no significant change occurred in the duration of Contract Negotiation from baseline to w/still in process clinical trials added. The t-test was used to compare differences between groups.
